# Development and testing of an electronic frailty index using Canadian electronic medical record data in primary care

**DOI:** 10.1186/s12875-025-03075-7

**Published:** 2025-11-12

**Authors:** Manpreet Thandi, Andy Gibb, Morgan Price, Jennifer Baumbusch, Sabrina T. Wong

**Affiliations:** 1 School of Nursing, University of British Columbia, T201 2211 Wesbrook Mall, Vancouver, BC V6T 2B5 Canada; 2 Centre for Health Services and Policy Research, University of British Columbia, 201-2206 East Mall, Vancouver, BC V6T 1Z3 Canada; 3 Department of Family Practice, University of British Columbia, David Strangway Building, Suite 300 5950 University Boulevard, Vancouver, BC V6T 1Z3 Canada

**Keywords:** Frailty, Frailty screening, Aging, Primary care, Polypharmacy, Cognitive impairment

## Abstract

**Background:**

Frailty is a state of increased vulnerability from physical, social, and cognitive factors and can result in several negative health outcomes at an individual and systemic level. Existing electronic medical record (EMR) data can be optimized to identify patients’ frailty level in primary care to facilitate early intervention and management of frailty in an efficient manner. The purpose of this work was to develop and validate a Canadian electronic frailty index (eFI) using primary care EMR data.

**Methods:**

We built a Canadian eFI based on the existing UK 36-factor eFI and tested it using EMR data from British Columbia (BC) primary care practices. We used a retrospective cross-sectional design to examine the concurrent criterion validity of the eFI by testing the hypotheses that increasing frailty is associated with (1) higher numbers of primary care visits, (2) increased presence of polypharmacy, and (3) increased presence of cognitive impairment. Hypotheses were tested using Poisson and Logistic regression modelling. The data source for analysis was the BC-Canadian Primary Care Sentinel Surveillance Network.

**Results:**

Our frailty algorithm was successful in its ability to calculate frailty scores for patients. A total of 15,178 patients met eligibility criteria from 22 primary care practices and 108 care providers. Ages ranged from 65 to 109 (mean 75.7); 54.2% were females. The number of frailty factors detected for patients ranged from 0 to 28 (mean 7.1). Analyses showed significant associations (*p* < 0.0001) between frailty levels and increasing age, material deprivation, and social deprivation. There were significant associations (*p* < 0.0001) between increasing frailty scores and our three outcomes. Individuals who were severely frail had nine more annual primary care visits, nine times the odds of concurrent polypharmacy, and approximately double the odds of cognitive impairment than someone who was not frail.

**Conclusions:**

Our study provides evidence for initial implementation of the eFI in primary care. There is significant potential for EMR data to facilitate early detection of frailty and drive care planning with healthcare teams. Integrating the eFI within primary care provides a tremendous opportunity to screen and manage frailty with the long-term goal of reducing negative patient health outcomes and often unnecessary healthcare costs.

**Supplementary Information:**

The online version contains supplementary material available at 10.1186/s12875-025-03075-7.

## Background

Frailty, a state of increased vulnerability from physical, social, and cognitive factors, often results in a greater risk of negative health-related individual and system outcomes [1–4].

Frailty leads to functional decline, decreased quality of life, and loss of independence; systemically, frailty results in increased rates of hospitalizations, long-term care admissions, and premature mortality [2, 4–7]. Moreover, Lavado et al. [8] report an additional healthcare cost of 2.25-fold for frail compared to non-frail patients.

Some of these additional costs related to frailty may be avoidable by reversing the condition with appropriate and timely interventions [9–12]. Assessing for frailty early in patients’ health trajectories in primary care settings has the potential to prevent longer term negative health outcomes. However, despite multiple existing frailty identification tools [13], time constraints and competing demands of practice are two of the most significant reasons for limited frailty assessments in primary care [14, 15].

An efficient and consistent approach is necessary to identify individuals who are frail or at risk of frailty [16]. With 93% of primary care practices in Canada [17] and 88% in the USA [18] now using electronic medical records (EMRs), and the uptick in use of digital health, there is high potential to use EMR data in primary care to identify frailty early and work with patients to maintain or improve their functional state. Past work [19] suggests that an automated frailty screening tool can use existing EMR data to calculate frailty scores and support further clinical decision-making.

The UK 36-factor electronic frailty index (eFI) [19] was originally developed to address some of the shortcomings of existing frailty assessments. It is based on the accumulation of deficits and calculates frailty scores for patients using routinely collected data that already exist in patients’ EMRs. The eFI is used across 99% of UK primary care settings, has been standardized as part of nationwide guidelines for elderly care across the National Health Service (NHS) [20], and is validated by several authors [21–26], demonstrating its usefulness and applicability in practice. A key strength of the eFI is that clinical terminologies representing frailty deficits can be mapped to international contexts.

The purpose of our work was to develop and validate a Canadian electronic frailty index using primary care EMR data. Our specific research question was: Does the criterion validity of the eFI demonstrate evidence to support its implementation in British Columbia (BC) primary care settings? We tested three hypotheses to examine the criterion validity of our eFI:

1. Higher eFI scores are associated with higher numbers of primary care visits.
2. Higher eFI scores are associated with an increased presence of polypharmacy.
3. Higher eFI scores are associated with an increased presence of cognitive impairment.

## Methods

We used a cross-sectional study design and a psychometric analytic framework. We first built a Canadian eFI and then tested it using EMR data from Canadian primary care practices.

This study was phase three of a larger research project [15, 27] and built upon the UK 36-factor eFI.

### Previous work

In phase one [15], we completed a modified Delphi study to determine whether BC primary care clinicians and older adults believed that frailty was adequately represented in the UK eFI. Thirty-three (92%) of the frailty factors achieved consensus by a diverse panel of Delphi participants (family physicians, nurse practitioners, nurses, allied health team members, older adults), providing content validation of the tool. This was the first study to review the conceptualization of frailty before translation to another context.

In phase two [27], we mapped the 36 factors representing frailty in the UK eFI to standardized terminologies used in Canadian primary care. Clinical terminologies were derived from: (a) diagnostic codes (from ICD9 [28], and ICD9-CM [29]); (b) laboratory tests/results (from LOINC [30]); and 3) medication prescriptions (ATC codes [31]). Polypharmacy was determined by whether there were five or more unique medications documented in the patient’s EMR in the past 12 months. We also developed a list of free text terms based on a sample of 527,521 EMR data entries. A total of 3,768 terms were identified to reflect the 36 frailty factors; 3,021 were standardized codes and 747 were free text terms (available as supplemental material).

### Data source

We used EMR data collected by the British Columbia – Canadian Primary Care Sentinel Surveillance Network (BC-CPCSSN) [32]. BC-CPCSSN consists of data from 111 primary care providers, and 136,146 patients as of June 30, 2022. BC-CPCSSN extracts de-identified and anonymized patient data from EMR systems and standardizes these data into a common format. Detailed information about the data is provided in Appendix Table 1.

### Procedures

#### Part 1: development of frailty algorithm

##### Foundation of the frailty algorithm

The 3,768 terms reflecting frailty factors form the foundation of the frailty algorithm (see supplemental material). A table listing each of the 36 frailty factors and the associated number of clinical terminologies is provided in Appendix Table 2.

##### Creation of queries for frailty algorithm

We worked with a data analyst to build a series of queries that make up the frailty algorithm. For each frailty factor, a query was created using structured query language (SQL), a programming language for storing and processing information in a database. The queries were built to look in the following EMR data tables: billing, encounter diagnosis, health condition, lab (test and result), medication, and referral.

Sometimes specific criteria were added to the free text queries to ensure the correct terms were being captured (Appendix Table 3). For example, to capture “home visit” but not “care home visit,” we needed to write the algorithm in the form [“home visit” AND NOT LIKE “care home visit”]. A table showing all free text terms and how they were captured within the algorithm is available as supplemental material.

In addition to the 36 queries built for each frailty factor, six additional queries were created for us to successfully execute the frailty algorithm, populate relevant tables, and calculate frailty scores (Appendix Table 4).

##### Execution of frailty algorithm and exporting of relevant data

We executed 42 queries on our dataset using the Database (DB) Browser for SQLite, a program that allows for searching large databases and exporting output. Queries were applied to patients who met the inclusion criteria (≥ 65 years, visited primary care at least once in the last two years, data collected between July 1, 2012 and June 30, 2022). Our criteria for patient inclusion was based on the CPCSSN recommendations of a 2 year contact group to define a denominator for research [33].

#### Part 2: testing the algorithm

We used a retrospective cross-sectional design to examine the concurrent criterion validity of the eFI by assessing whether our frailty algorithm is able to demonstrate a relationship between frailty scores and common outcomes associated with frailty. Based on existing research, we had three hypotheses:

1. Higher eFI scores are associated with higher numbers of primary care visits*.* Previous research has shown that increasing levels of frailty are associated with increased healthcare system use [34–38].
2. Higher eFI scores are associated with an increased presence of polypharmacy. Past work suggests that increasing frailty is associated with increased presence of polypharmacy [39–41].
3. Higher eFI scores are associated with an increased presence of cognitive impairment. Past work suggests that increasing frailty is associated with increased presence of cognitive impairment [42–47].

### Independent variable

Our independent variable of interest was the eFI score. Based on their number of frailty factors, patients were placed into one of four categories: fit (0–4 factors), mild frailty (5–8 factors), moderate frailty (9–12 factors), or severe frailty (>12 factors) [19]. These categories align with the UK eFI frailty categories, which were based on the Rockwood Clinical Frailty Scale [4, 48] (Appendix Table 5).

### Dependent variables

1. *Primary Care Visits* (count variable). The number of primary care visits in the last 12 months was identified from the extracted EMR data.
2. *Polypharmacy* (binary variable). To test the association between frailty and polypharmacy, we used the definition of concurrent or simultaneous polypharmacy [49–51]. This meant having five or more medication prescriptions documented *simultaneously* in the last 12 months. Because we did not have a specific query that can identify which patients meet the concurrent polypharmacy definition in a large dataset, additional steps were required using Python Software and data available from BC-CPCSSN (see supplemental material).
3. *Cognitive Impairment* (binary variable). Patients who received a cognitive impairment flag were those with least one code or free text term indicating cognitive impairment in the EMR.

### Potential confounding variables

Potentially confounding variables of interest included: age, sex, rural/urban status, material and social deprivation level. Rural/urban status and material/social deprivation levels were constructed using patients’ postal codes. Values for deprivation levels are related to the Pampalon Index [52, 53] and range from 1 (least deprived) to 5 (most deprived). Material deprivation scores indicate the consequences of a lack of material resources associated with low education, insecure employment situation, and insufficient income [54]. The social deprivation score refers to a fragile social network; it includes living alone, being a single parent, or being separated/divorced/widowed [54].

Deprivation indices are often used as a proxy for information on socioeconomic status [54]. The values represent the percentage of people within a specific postal code that have each score, and not an individual patient’s deprivation score. For example, within a patient’s postal code, 10% of people could have social deprivation = 1, 40%=2, 50%=3, 0%=4, 0%=5 [50]. Thus, only when 100% of people in a postal code had a specific deprivation score, was the score assigned to the individual patient. The remaining patients were considered to have missing data for deprivation level.

## Analyses

### Part 1: patient sample & algorithm output

We completed descriptive summary statistics to report on patient characteristics (age, sex, geographic area, deprivation scores), proportions of patients in each frailty category, number of frailty factors possessed by patients, and the most frequently identified frailty factors. These analyses were completed using Microsoft Excel.

We also completed inferential statistical tests to examine relationships between mean frailty levels and patient characteristics using the Analysis of Variance (ANOVA) statistical test and follow up Tukey’s Honest Significant Difference (HSD) Tests as necessary. These analyses were completed using R Statistical Software [55].

### Part 2: associations between frailty and primary care visits, polypharmacy, and cognitive impairment

**Fig. 1:**
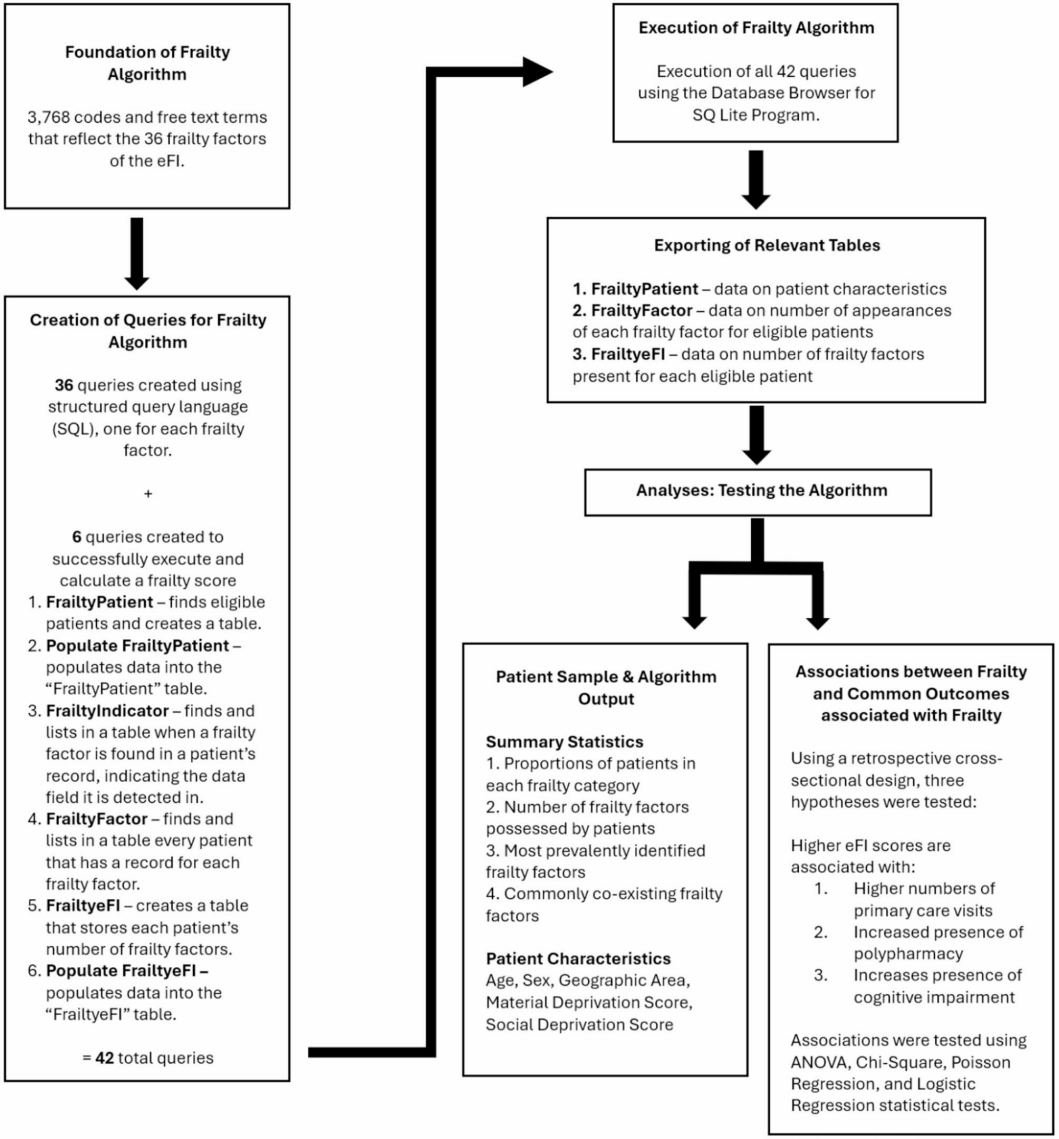
Summary of methods

For missing sex, geographic area, material and social deprivation level data, multiple imputation was completed using R Software. Multiple imputation is a simulation-based statistical technique for handling missing data where missing values were identified and replaced by a random sample of plausible value imputations [56]. This step addresses the potential bias that would otherwise be introduced by including only complete cases. We used ANOVA and Chi-square analyses to test initial associations. To control for potentially confounding variables, we conducted Poisson and Logistic regression modeling. Table 1 provides a summary of these analyses. Fig. 1 provides a summary of the methods used in this study.

**Table 1:** Summary of analyses

Independent Variable (Type)	Dependent Variable (Type)	Statistical Test(s) Performed	Potential Confounding Variables Adjusted for in Regression Analyses
eFI Score (categorical)	Primary care visits (discrete/count)	Analysis of Variance (ANOVA) Poisson Regression	Age Sex Urban/Rural Setting Material Deprivation Level Social Deprivation Level
eFI Score (categorical)	Polypharmacy (binary)	Chi Square Logistic Regression	
eFI Score (categorical)	Cognitive impairment (binary)	Chi Square Logistic Regression	

All analyses were completed using R Statistical Software.

### Ethical considerations

This study was approved by the University of British Columbia Behavioural Research Ethics Board (H22-00692). To access the BC-CPCSSN data repository, a data access request was requested and granted. All data were accessed through Population Data BC’s [57] secure research environment and contained no patient identifying information.

CPCSSN has its own ethics approval from the University of British Columbia Behavioral Ethics Board. It was not necessary to obtain informed consent from patients whose data was accessed through CPCSSN as patient EMR data are already routinely collected through CPCSSN.

## Results

### Part 1: patient sample & algorithm output

Our frailty algorithm was successful in its ability to calculate frailty scores by providing a count of the number of frailty factors present for each patient. Dividing the number of frailty factors present by the total number of frailty factors provides a continuous frailty index score [19].

We aimed to capture those patients who are most likely to access and use primary care services.

A total of 15,178 patients met the eligibility criteria from 22 BC primary care practices and 108 care providers. As long as a patient’s most responsible practitioner is participating in CPCSSN, they were included regardless of where they live. There were 6,920 (45.6%) males and 8,227 (54.2%) females. Ages ranged from 65 to 109 (mean 75.7, SD 8.1). Only 6% of the patients lived in a rural setting compared to 87% living in an urban setting. Missing data for patients’ geographical location was due to missing postal code information. Table 2 provides a summary of patient characteristics.

**Table 2:** Patient characteristics (*N* = 15,178)

Characteristics	Overall, n(%)	Fit Group	Mild Frailty Group	Moderate Frailty Group	Severe Frailty Group
Frailty Group, n(%)	15,178	4,534 (29.9)	5,477 (36.1)	3,267 (21.5)	1,900 (12.5)
Age					
Mean age (SD)	75.7 (8.1)	73.4 (7.6)	75.1 (7.6)	77.5 (8.2)	79.8 (8.6)
Median age (range)	74.0 (65–109)	71.0 (65–107)	74.0 (65–107)	76.0 (65–109)	79.0 (65–106)
Age (years), n(%)					
65–69	4,105 (27.0)	1,743 (38.4)	1,501 (27.4)	613 (18.8)	248 (13.1)
70–74	3,769 (24.8)	1,241 (27.3)	1,480 (27.0)	725 (22.2)	323 (17.0)
75–79	2,988 (19.7)	749 (16.5)	1,115 (20.3)	733 (22.4)	391 (20.6)
80–84	1,940 (12.8)	363 (8.0)	690 (12.6)	530 (16.2)	357 (18.8)
85–89	1,208 (8.0)	191 (4.2)	383 (7.0)	350 (10.7)	284 (14.9)
>90	1,168 (7.7)	247 (5.4)	308 (5.6)	316 (9.7)	297 (15.6)
Sex, n(%)					
Male	6,920 (45.6)	2,080 (45.9)	2,519 (46.0)	1,504 (46.0)	817 (43.0)
Female	8,227 (52.2)	2,440 (53.8)	2,947 (53.8)	1,760 (53.9)	1,080 (56.8)
Geographic Area, n(%)					
Urban	13,153 (86.7)	3,665 (80.8)	4,843 (88.4)	2,919 (89.3)	1,726 (90.8)
Rural	919 (6.1)	348 (7.7)	298 (5.4)	185 (5.7)	88 (4.6)
Material Deprivation Score, n(%)					
1 (least deprived)	2,731 (18.0)	883 (19.5)	990 (18.1)	539 (16.4)	319 (16.8)
2	2,261 (14.9)	674 (14.9)	852 (15.6)	480 (14.7)	255 (13.4)
3	2,119 (14.0)	548 (12.1)	792 (14.5)	476 (14.6)	303 (15.9)
4	1,693 (11.2)	431 (9.5)	610 (11.1)	404 (12.4)	248 (13.1)
5 (most deprived)	1,299 (8.6)	295 (6.5)	459 (8.4)	342 (10.5)	203 (10.7)
Mean Material Deprivation Score (SD)	2.7 (1.4)	2.5 (1.3)	2.6 (1.4)	2.8 (1.4)	2.8 (1.4)
Social Deprivation Score, n(%)					
1 (least deprived)	1,386 (9.1)	418 (9.2)	538 (9.8)	302 (9.2)	128 (6.7)
2	1,738 (11.5)	544 (12.0)	681 (12.4)	338 (10.3)	175 (9.2)
3	1,713 (11.3)	500 (11.0)	646 (11.8)	374 (11.4)	193 (10.1)
4	2,673 (17.6)	687 (15.1)	948 (17.3)	651 (20.0)	387 (20.4)
5 (most deprived)	2,622 (17.3)	698 (15.4)	891 (16.3)	594 (18.2)	439 (23.1)
Mean Social Deprivation Score	3.3 (1.4)	3.2 (1.4)	3.3 (1.4)	3.4 (1.4)	3.6 (1.3)

*Missing Data: Material Deprivation: 33.4% (n=5,075); Social Deprivation: 33.2% (n=5,046); Geographic Area: 7.3% (n=1,106); Sex: 0.2% (n=31)

The number of frailty factors detected for patients ranged from 0 to 28 (mean 7.1, SD 4.3). A total of 14,886 (98.1%) of patients had at least one frailty factor. A total of 4,658 (30.7%) of patients were categorized as “fit” (0–4 frailty factors). Table 3 shows the number of patients and their associated number of frailty factors. The top ten frailty factors identified were (in order of decreasing frequency): hypertension, arthritis, urinary system disease, chronic kidney disease, anaemia/haematinic deficiency, diabetes, respiratory disease, polypharmacy (cumulative), ischemic heart disease, and thyroid disorder.

**Table 3:** Number of patients of associated number of frailty factors

Number of Frailty Factors	Number of Patients
0	292
1	665
2	1062
3	1247
4	1392
5	1417
6	1479
7	1368
8	1263
9	983
10	899
11	707
12	601
13	458
14	364
15	295
16	202
17	144
18	108
19	83
20	61
21	32
22	21
23	14
24	11
25	6
26	0
27	3
28	1

Table 4 provides a summary of frailty score distribution by age, sex, geographic area, and deprivation levels. Analyses showed significant associations between frailty level and increasing age (df = 5, F = 254.8, *p* < 0.0001), material deprivation (df = 4, F = 19.23, *p* < 0.0001), and social deprivation (df = 4, F = 22.07, *p* < 0.0001).

**Table 4:** Summary of eFI distribution

	Overall, n(%)	Fit Group	Mild Frailty Group	Moderate Frailty Group	Severe Frailty Group
Frailty Group, n(%)	15,178	4,534 (29.9)	5,477 (36.1)	3,267 (21.5)	1900 (12.5)
n=15,178					
Frailty Score					
Mean # factors (SD)	7.1 (4.3)	2.6 (1.2)	6.5 (1.1)	10.3 (1.1)	15.5 (2.6)
Mean eFI (SD)	0.2 (0.1)	0.1 (0.03)	0.2 (0.03)	0.3 (0.03)	0.4 (0.1)

	Number of Frailty Factors, mean (SD)	Frailty Index Score, mean (SD)	Significance
Frailty by Age (years)			p<0.0001*
65–69 (ref)	5.8 (3.8)	0.2 (0.1)	
70–74	6.7 (4.0)	0.2 (0.1)	*
75–79	7.6 (4.2)	0.2 (0.1)	*
80–84	8.5 (4.4)	0.2 (0.1)	*
85–89	9.3 (4.8)	0.3 (0.1)	*
>90	9.2 (5.2)	0.3 (0.1)	*
Frailty by Sex			
n=15,147			p=0.115
Male	7.2 (4.4)	0.2 (0.1)	
Female	7.3 (4.4)	0.2 (0.1)	
Frailty by Geographic Area			
n=14,072			p<0.0001*
Urban	7.4 (4.4)	0.2 (0.1)	
Rural	6.7 (4.4)	0.2 (0.1)	
Frailty by Material Deprivation Score			
n=10,103			p<0.0001*
1 (least deprived) (ref)	7.1 (4.4)	0.2 (0.1)	
2	7.1 (4.2)	0.2 (0.1)	
3	7.7 (4.4)	0.2 (0.1)	*
4	7.8 (4.4)	0.2 (0.1)	*
5 (most deprived)	8.1 (4.5)	0.2 (0.1)	*
Frailty by Social Deprivation Score			
n=10,132			p<0.0001*
1 (least deprived) (ref)	7.0 (4.2)	0.2 (0.1)	
2	6.9 (4.1)	0.2 (0.1)	
3	7.2 (4.1)	0.2 (0.1)	
4	7.7 (4.4)	0.2 (0.1)	*
5 (most deprived)	7.9 (4.7)	0.2 (0.1)	*

*Ref* Reference group

*statistically significant association between group and reference group

### Part 2: associations between frailty and primary care visits, polypharmacy, and cognitive impairment

**Fig. 2:**
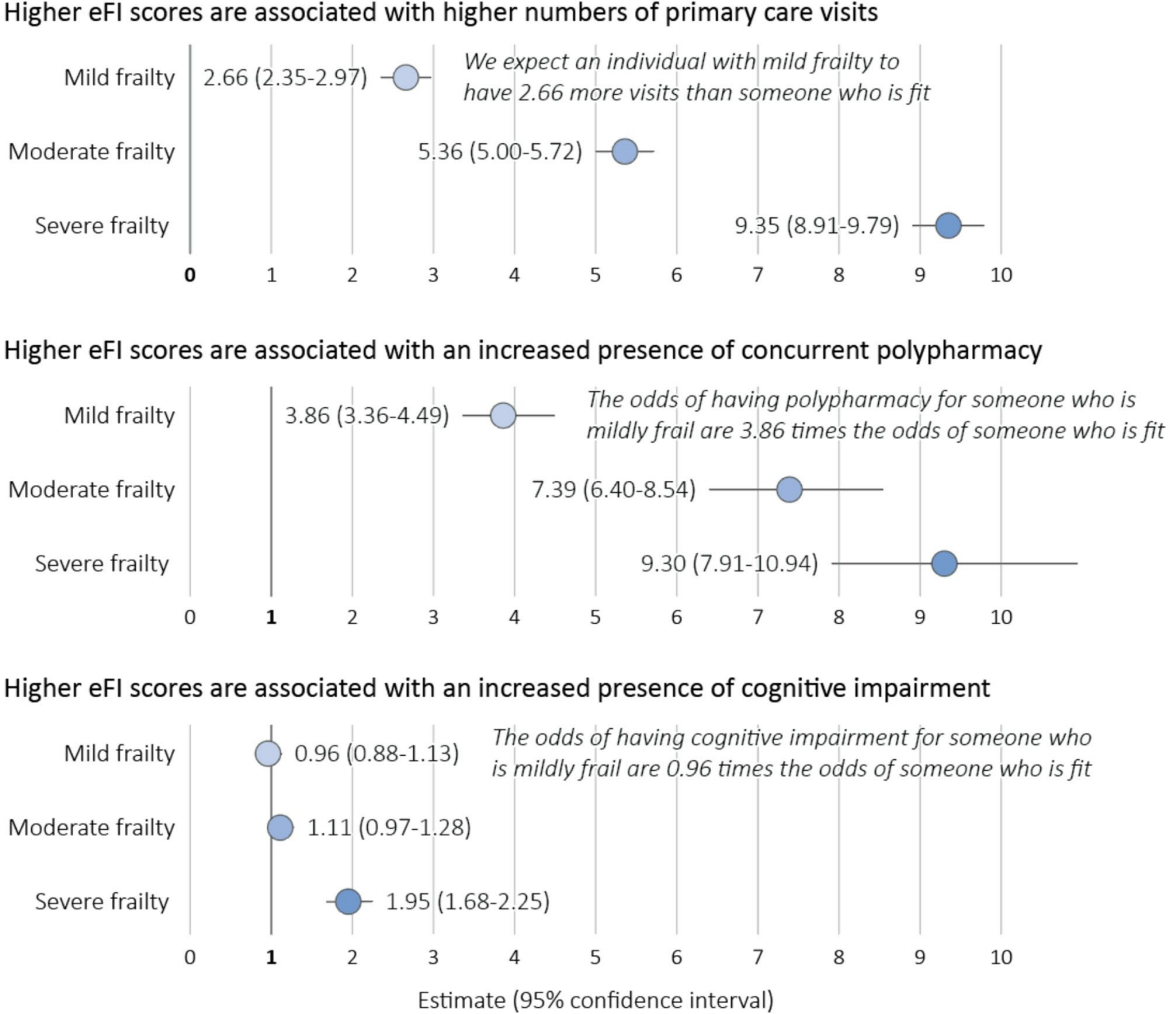
Summary of hypotheses testing results

We recognize that polypharmacy and cognitive impairment are frailty factors and included in the calculation of the eFI score. Thus, to account for any overlap or correlation, we removed these two factors from the frailty score calculation for hypotheses testing. All results are summarized in Table 5. Fig. 2 depicts these results in graphical form.

**Table 5:** Summary of regression analyses for hypotheses 1, 2, and 3

**Hypothesis** 1: Higher eFI scores are associated with higher numbers of primary care visits. **Statistical Test**: Poisson Regression **Confounding Variables & Association with Outcome Variable**: Age (*p* < 0.0001), rural/urban status (*p* < 0.0001), material deprivation (*p* = 0.0029), social deprivation (*p* < 0.0001). The variable sex was not statistically associated with the outcome variable (*p* = 0.983) and did not change the results of the analysis, thus was not considered a confounding variable.						
**Frailty Category**	**Mean (SD)[IQR] number of primary care visits in last 12 months**	**Effect Size Estimate**	**Standard Error**	**95% Confidence Interval**	**Significance Level**	**Interpretation**
Fit (ref) (*n* = 4534)	3.9 (4.6) [1,5]	-	-	-	-	-
Mild Frailty (*n* = 5477)	6.4 (6.8) [3,8]	2.66	0.16	2.35–2.97	*p* < 0.0001	We expect an individual with mild frailty to have **2.66** more visits than someone who is fit
Moderate Frailty (*n* = 3267)	8.9 (8.8) [4,11]	5.36	0.18	5.00–5.72	*p* < 0.0001	We expect an individual with moderate frailty to have **5.36** more visits than someone who is fit
Severe Frailty (*n* = 1900)	12.8 (13.4) [6,16]	9.35	0.22	8.91–9.79	*p* < 0.0001	We expect an individual with severe frailty to have **9.35** more visits than someone who is fit
**Clinical Significance**: There are clinically significant associations between frailty levels and number of primary care visits across all frailty groups. There is no overlap in confidence intervals across any of the frailty groups.						
**Hypothesis** 2: Higher eFI scores are associated with an increased presence of concurrent polypharmacy. **Statistical Test**: Logistic Regression **Confounding Variables & Association with Outcome Variable**: Age (*p* < 0.0001), sex (*p* = 0.04), material deprivation (*p* < 0.0001), social deprivation (*p* < 0.0001). The variable urban/rural status was not statistically associated with the outcome variable (*p* = 1) and did not change the results of the analysis, thus was not considered a confounding variable.						
**Frailty Category**	**Prevalence of Concurrent Polypharmacy**	**Effect Size Estimate**	**Standard Error**	**95% Confidence Interval**	**Significance Level**	**Interpretation**
Fit (ref) (*n* = 4774)	5.9%	-	-	-	-	-
Mild Frailty (*n* = 5681)	20.0%	3.86	0.07	3.36–4.49	*p* < 0.0001	The odds of having polypharmacy for someone who is mildly frail is **3.86** times the odds of someone who is fit.
Moderate Frailty (*n* = 3107)	32.5%	7.39	0.07	6.40–8.54	*p* < 0.0001	The odds of having polypharmacy for someone who is moderately frail is **7.39** times the odds of someone who is fit.
Severe Frailty (*n* = 1616)	37.4%	9.30	0.08	7.91–10.94	*p* < 0.0001	The odds of having polypharmacy for someone who is severely frail is **9.30** times the odds of someone who is fit.
**Clinical Significance**: There are clinically significant associations between frailty levels and presence of polypharmacy across all frailty groups. There is no overlap in confidence intervals for mild and moderate frailty levels and little overlap between moderate and severe frailty levels.						
**Hypothesis** 3: Higher eFI scores are associated with an increased presence of cognitive impairment. **Statistical Test**: Logistic Regression **Confounding Variables & Association with Outcome Variable**: Age (*p* < 0.0001), urban/rural status (*p* < 0.0001), material deprivation (*p* = 0.0004), social deprivation (*p* < 0.0001). The variable sex was not statistically associated with the outcome variable (*p* = 0.5531) and did not change the results of the analysis; thus was not considered a confounding variable.						
**Frailty Category**	**Prevalence of Cognitive Impairment**	**Effect Size Estimate**	**Standard Error**	**95% Confidence Interval**	**Significance Level**	**Interpretation**
Fit (ref) (*n* = 4694)	12.2%	-	-	-	-	-
Mild Frailty (*n* = 5486)	13.3%	0.96	0.06	0.88–1.13	*p* = 0.948	The odds of having cognitive impairment for someone who is mildly frail is **0.96** times the odds of someone who is fit.
Moderate Frailty (*n* = 3211)	18.1%	1.11	0.07	0.97–1.28	*p* = 0.123	The odds of having cognitive impairment for someone who is moderately frail is **1.11** times the odds of someone who is fit.
Severe Frailty (*n* = 1787)	31.7%	1.95	0.07	1.68–2.25	*p* < 0.0001	The odds of having cognitive impairment for someone who is severely frail is **1.95** times the odds of someone who is fit.
**Clinical Significance**: There are clinically significant associations between frailty levels and presence of cognitive impairment for the moderate and severe frailty groups.						

#### Hypothesis 1: higher eFI scores are associated with higher numbers of primary care visits

The mean number of primary care visits for all patients was 7.0 (SD 8.4; IQR 2,9). The mean number of primary care visits increased for each level of frailty (3.9, 6.4, 8.9, and 12.8, respectively). Regression modeling showed statistically significant associations between frailty level and mean number of visits for all frailty groups. Relative to the fit group, individuals were expected to have 2.66 (mild frailty), 5.36 (moderate frailty), and 9.35 (severe frailty) more primary care visits.

#### Hypothesis 2: higher eFI scores are associated with an increased presence of polypharmacy

The total prevalence of concurrent polypharmacy was 20.0%. The prevalence increased across fit, mild, moderate, and severe frailty levels (5.9%, 20.0%, 32.5%, and 37.4%, respectively). Regression modeling showed a statistically significant association between frailty level and polypharmacy for all frailty groups. Relative to the fit group, the odds of concurrent polypharmacy were 3.86, 7.39, and 9.30 times higher for the mild, moderate, and severe frailty groups respectively.

#### Hypothesis 3: higher eFI scores are associated with an increased presence of cognitive impairment

The total prevalence of cognitive impairment was 16.2%. The prevalence increased across fit, mild, moderate, and severe frailty levels (12.2%, 13.3%, 18.1%, and 31.7%, respectively). Regression modeling showed a significant association between frailty level and cognitive impairment between the fit and severe frailty groups. The odds of cognitive impairment were 1.95 times higher for the severe frailty group.

## Discussion

We developed and tested an eFI using BC primary care data based on the UK 36-factor eFI. Our eFI consists of 3,768 codes and free text terms relevant to BC primary care settings that reflect the 36 factors of the eFI. This was the first study to our knowledge to develop a Canadian eFI. The eFI uses existing EMR data to calculate continuous frailty scores (rather than a binary frail/not frail approach), allowing frailty to be considered on a trajectory and providing an opportunity for early intervention and management. The use of existing data also mitigates the significant barrier of time constraints in primary care frailty screening. We discuss our findings within the context of three main messages.

### Our study provides evidence for initial implementation of the eFI in primary care

We developed and successfully tested an eFI using BC primary care data. Our cross-sectional analyses provide evidence of the eFI being associated with our three tested outcomes, aligning with existing literature: (1) Increased numbers of primary care visits [34–38]; (2) Polypharmacy [39–41]; and (3) Cognitive impairment [42–47]. To our knowledge, this is the first study examining concurrent polypharmacy and its association with frailty. Concurrent polypharmacy provides a stricter definition of polypharmacy than cumulative polypharmacy, further emphasizing this clear association. We note that the removal of the frailty factors polypharmacy and cognitive impairment from the frailty score calculation for hypotheses testing did not impact the results. We tested the model’s validation with and without the inclusion of these two factors and found very similar results, indicating a clear relationship between increasing frailty and increasing levels of polypharmacy and cognitive impairment.

Trends related to frailty’s associations with increasing age, social deprivation, and material deprivation in our study also align with existing literature [58–61]. Testing our eFI algorithm against these common frailty outcomes contributed to the validity that our algorithm worked as intended and supports its’ implementation in primary care settings for pilot testing. The statistically and clinically significant associations between frailty scores and our outcomes further emphasize the importance of needing to implement a frailty screening tool within primary care to identify frailty early and intervene to prevent unnecessary negative health outcomes.

### Future work can validate the eFI further

Future work would need to test and evaluate the eFI further. This would involve collaborating with primary care providers and EMR vendors to determine the best and most efficient way to integrate the eFI within EMR systems. We would also need to establish follow-up guidelines or care pathways for patients belonging to each frailty category.

We discuss potential approaches to further evaluate/test the eFI. First, looking at the eFI’s predictive ability. We did not link our data to administrative data in this study, thus we were unable to evaluate the predictive validity of higher frailty scores being associated with longer term outcomes such as hospitalizations, nursing home admissions, and mortality which has been done in other studies [19, 25, 62]. Past work suggests that higher frailty is indeed related to increased hospitalizations, increased nursing home admissions, and increased mortality rates [19, 25, 62]. Second, we can compare patient outcomes in clinics with the eFI implemented and those without using controlled studies. Assuming there are follow-up guidelines following an eFI score calculation, comparing patient outcomes in clinics with and without the ability to calculate an eFI score would emphasize the importance and significance of early identification in treating, reversing, or delaying frailty. Third, we can compare patient outcomes for those receiving intervention, specifically through the use of baseline and periodic measurements of frailty levels. Fourth, we can also assess the construct validity of the eFI by examining the convergent validity of the eFI with other common tools used to assess for and identify frailty. Finally, we could also evaluate the adoption and acceptance of a new tool/piece of technology. For example, the technology acceptance model (TAM) [63] examines four major variables when implementing new technology: perceived usefulness, perceived ease of use, behavioural intention, and actual behaviour, all of which are influenced by external behaviours. This approach can help assess primary care clinicians’ attitudes and perceptions of the eFI and whether it is being used as intended.

### EMRs in primary care hold significant potential and can be better optimized

Although there have been substantial increases in the use of EMRs in primary health care settings, the use of advanced features is limited, especially in Canada [64]. The most common uses of the EMR include checking patient notes, medication prescription and checking drug interactions, diagnosis management, billing, documentation, and electronic reminders [65]. There is significant potential for EMR use beyond these routine tasks, specifically frailty screening and management as part of routine care. Our eFI algorithm is able to provide frailty scores for patients using readily available EMR data. Implementing this algorithm into EMR systems will allow for early detection of frailty and care planning within healthcare teams.

### Limitations

The data that we used to build the eFI was limited to BC to keep the study within the scope of this research project. However, we searched a total of 527,521 data entries (previous work) to capture free text terms associated with frailty factors and data were derived from 15,178 patients from 22 BC primary care practices and 108 different primary care providers. Additionally, the use of ICD9 and ICD9-CM codes is not limited only to the province of BC, but across Canada, thus this tool could be applied on a pilot basis across Canada. We note that our sample is skewed toward urban patients (87% urban vs. 6% rural) which limits generalizability to rural Canadian settings and patients who may face greater healthcare access challenges and greater levels of social and/or material deprivation. However, it is likely a safe assumption that patients seen in primary care are representative of those who have access to and use primary care services, which reflects the target population of the eFI. Further research would need to be undertaken to engage with rural areas in the validation of the eFI.

We were able to use the definition of concurrent polypharmacy when evaluating polypharmacy as an outcome variable, however the definition of polypharmacy used in the eFI reflects cumulative polypharmacy. There are two reasons for this: our eFI is based on the UK 36-factor eFI and aligns with Clegg et al.’s [19] definition of polypharmacy, and we do not yet have a programming query that is able to extract concurrent polypharmacy in a large data set. Future work may be able to address this limitation.

We did not look at the quality of the EMR data within the scope of this study. Using a tool that relies on already existing EMR data would need to assume adequate data is being inputted into patients’ records. EMRs in Canada are predominantly used for billing purposes, making it easier to capture medical diagnoses and more difficult to capture syndromes, signs, or symptoms such as pain or confusion. We address this limitation in our preceding publication (10.1017/S1463423625000337) with the inclusion of free text data in the development of our frailty algorithm. Nonetheless, frailty scores would need to be interpreted under the assumption that clinicians have assessed and documented relevant issues related to frailty.

Finally, we did not confirm the presence or absence of frailty factors in patients’ records using chart reviews and/or manual calculation of eFI scores for comparison of results. Future research can address this limitation to further add to the validity of the eFI.

## Conclusions

This study was successful in its aim of developing a frailty screening algorithm usingexisting EMR data. Using existing EMR data to calculate frailty scores for patients provides a great opportunity to screen and manage frailty with the long-term goal ofreducing negative patient health outcomes and often unnecessary healthcare costs. This study was a first step towards frailty screening standardization in BC and potentially Canada. Using existing EMR data to calculate frailty scores for patients provides a great opportunity to efficiently and consistently screen and manage frailty with the long-term goal of reducing negative patient health outcomes and often unnecessary healthcare costs.

## Supplementary Information

Supplementary Material 1.

Supplementary Material 2.

Supplementary Material 3.

Supplementary Material 4.

## Data Availability

All data generated or analyzed during this study are included in this published article (and its supplementary information files). Any further requested information is available from the corresponding author on reasonable request.

## Abbreviations

ANOVA: Analysis of Variance
ATC: Anatomical Therapeutic Codes
BC: British Columbia
CPCSSN: Canadian Primary Care Sentinel Surveillance Network
DB: Database Browser
eFI: Electronic Frailty Index
EMR: Electronic Medical Record
HSD: Honest Significance Difference
ICD: International Classification of Diseases
ICD9-CM: International Classification of Diseases Clinical Modification
LOINC: Logical Observation Identifiers Names and Codes
NHS: National Health Service
SQL: Structured Query Language
TAM: Technology Acceptance Model
UK: United Kingdom
