# Supplementary material for: Development and testing of an electronic frailty index using Canadian electronic medical record data in primary care
Source: BMC Prim Care. 2025 Nov 12;26:359. doi: 10.1186/s12875-025-03075-7 (PMC12613384; doi:10.1186/s12875-025-03075-7)
Supplement: Supplementary file 1 — Supplementary Material 1. [file 12875_2025_3075_MOESM1_ESM.docx]

**Appendix**

**Appendix Table 1: CPCSSN Data Tables Accessed**

| **CPCSSN Data Table** | **Description** |
| --- | --- |
| EncounterDiagnosis | Diagnoses resulting from each encounter between patient and provider |
| Encounter | Encounters/interactions between patient and provider |
| Patient | Contains demographic information for each patient |
| Billing | Contains patient-level EMR billing records processed by CPCSSN |
| HealthCondition | Problem list and medical history of the patient |
| Lab | Results of lab tests |
| Medication | Medications prescribed for the patient |
| Referral | All referrals made by their assigned provider or clinic |
| Site | Contains general information on clinic location and type |
| Cycle | Contains date information for the data cut |

*Data access was granted to EMR data meeting the following criteria: charts between July 1, 2012, and June 30, 2022, for patients aged ≥65 years with a primary care visit within the last 24 months. Access to the following data tables and variables was granted: “EncounterDiagnosis”, “Encounter”, “Patient”, “Billing”, “HealthCondition”, “Lab”, “Medication”, “Referral”, “Site,” and “Cycle.” These data tables contained all clinical terminologies that reflect the 36 frailty factors (i.e. clinical codes, lab results, medications, and free text) of the eFI.*

**Appendix Table 2: 36 frailty factors of the eFI used to build frailty algorithm**

| **Frailty Factors** | **Total Number of Codes/Free Text Terms** |
| --- | --- |
| Activity Limitation  Anaemia and Haematinic Deficiency  Arthritis  Atrial Fibrillation  Cerebrovascular Disease  Chronic Kidney Disease  Diabetes  Dizziness  Dyspnea  Falls  Foot Problems  Fragility Fracture  Hearing Impairment  Heart Failure  Heart Valve Disease  Housebound  Hypertension  Hypotension/Syncope  Ischemic Heart Disease  Memory and/or Cognitive Problems  Mobility and Transfer Problems  Osteoporosis  Parkinsonism and Tremor  Peptic Ulcer  Peripheral Vascular Disease  Polypharmacy  Requirement for Care  Respiratory Disease  Skin Ulcer  Sleep Disturbance  Social Vulnerability  Thyroid Disorder  Urinary Incontinence  Urinary System Disease  Visual Impairment  Weight Loss and/or Anorexia | 33  109  246  23  122  83  126  49  22  45  97  851  58  24  44  15  61  41  107  77  67  12  17  139  102  *  21  126  47  95  88  75  33  165  518  29 |
| Total: | 3,768 |

** Polypharmacy was defined as having five or more unique medication prescriptions documented in the patient’s primary care CPCSSN record over the last 12 months. We developed a query to detect number of medications documented.*

**Appendix Table 3: Criteria used to ensure capturing of correct terms by frailty algorithm**

| **Criteria** | **Description** | **Example** |
| --- | --- | --- |
| “NOT LIKE” | Used to specify exclusion criteria in free text terms | If. |
| **%** | Used to capture terms that can be matched anywhere in free text (i.e. nested terms) | The term “%B12 low%” would also capture “vitamin B12 low.” |
| LIKE and % | Used to capture subcodes within an ICD9 or ICD9-CM code | The term “LIKE ‘715%’” would also capture all subcodes under the code 715 |
| = | Used when we only wanted to capture a specific code without capturing its subcodes | “=715.3” would only capture this specific code and not sub-codes |
| Creation of a new term | Used when we wanted to capture multiple terms for efficiency of the algorithm | The new term “hypotensi” (used in conjunction with the % symbol; i.e. “%hypotensi%) captures the following terms: hypotension, hypotension chronic, hypotension orthostatic, hypotension persistent, hypotensive, and hypotensive episode. |

**Appendix Table 4: Additional queries required for execution of the frailty algorithm**

| **Query Name** | **Description** |
| --- | --- |
| **1. Create the necessary tables** | |
| Create FrailtyPatient | Used for storing details for patients meeting the eligibility criteria (65 years and older and at least one health care provider visit in the last 2 years) |
| Create FrailtyIndicator | Used for storing the reasons why a patient satisfies the criteria for a given factor, indicating the source of the code and/or free text term (i.e. which data field detected the presence of the code/free text). |
| Create FrailtyFactor | Used for storing a list of patients and their associated frailty factors. |
| Create FrailtyEFI | Used for storing a list of patients and their associated number of eFI factors. |
| **2. Populate the relevant tables** | |
| Populate FrailtyPatient | Populates data into the “FrailtyPatient” table including patient ID, sex, age at start of data time frame, and number of unique medications documented in the patient’s record (initially 0 and populated by the polypharmacy query). |
| Populate FrailtyEFI | Populates data into the “Frailty eFI” table. |

*For each factor, a query was executed that entered matching records into the FrailtyIndicator table and thence added entries to the FrailtyFactor table for patients with that factor. After all 36 queries have been executed, the FrailtyEFI table is populated with an entry for each eligible patient and the number of factors for that patient.*

**Appendix Table 5: eFI Categories (Clegg et al., 2016; NHS Scotland, 2019)**

| **Frailty Category** | **eFI Score** | **Description (Rockwood et al., 2005)** |
| --- | --- | --- |
| Fit | 0-0.12  (0-4 frailty factors) | Very Fit - People who are robust, active, energetic and motivated. These people commonly exercise regularly. They are among the fittest for their age.  Well - People who have no active disease symptoms but are less fit than category 1. Often, they exercise or are very active occasionally, e.g. seasonally.  Managing Well - People whose medical problems are well controlled but are not regularly active beyond routine walking. |
| Mild frailty | >0.12 – 0.24  (5-8 frailty factors) | Vulnerable - While not dependent on others for daily help, often symptoms limit activities. A common complaint is being “slowed up”, and/or being tired during the day.  Mildly Frail - These people often have more evident slowing, and need help in high order IADLs (finances, transportation, heavy housework, medications). Typically, mild frailty progressively impairs shopping and walking outside alone, meal preparation and housework. |
| Moderate frailty | >0.24 – 0.36  (9-12 frailty factors) | Moderately Frail - People need help with all outside activities and with keeping house. Inside, they often have problems with stairs and need help with bathing and might need minimal assistance (cuing, standby) with dressing. |
| Severe frailty | >0.36  (>12 frailty factors) | Severely Frail - Completely dependent for personal care, from whatever cause (physical or cognitive). Even so, they seem stable and not at high risk of dying (within ~ 6 months).  Very Severely Frail - Completely dependent, approaching the end of life. Typically, they could not recover even from a minor illness.  Terminally Ill - Approaching the end of life. This category applies to people with a life expectancy. |
