# Supplementary material for: Development and testing of an electronic frailty index using Canadian electronic medical record data in primary care
Source: BMC Prim Care. 2025 Nov 12;26:359. doi: 10.1186/s12875-025-03075-7 (PMC12613384; doi:10.1186/s12875-025-03075-7)
Supplement: Supplementary file 2 — Supplementary Material 2. [file 12875_2025_3075_MOESM2_ESM.docx]

This document includes tables with the free text terms used in the development of queries for each frailty factor along with added criteria to ensure correct terms were being captured.

**Activity Limitation**

| **Free Text Term** | **Notes** |
| --- | --- |
| debilitation |  |
| debility |  |
| deconditioning |  |
| deteriorating health |  |
| difficulty walking |  |
| disability |  |
| disability form | captured under disability |
| lower extremity weakness |  |
| general deconditioning |  |
| hard to walk |  |
| leg weakness |  |
| muscle wasting |  |
| parking permit |  |
| sarcopenia |  |
| trouble walking |  |
| weakness | exclusions: arm weakness, no weakness, weakness in arms, hand weakness, shoulder weakness |

**Anaemia and Haematinic Deficiency**

| **Free Text Term** | **Notes** |
| --- | --- |
| Anaemia |  |
| Anemia |  |
| B12 deficiency |  |
| B12 injection |  |
| B12 low |  |
| B12 shot |  |
| Ferritin low |  |
| Hgb low |  |
| Iron deficiency |  |
| Iron deficiency anemia | captured under iron deficiency |
| Iron deficiency anaemia | captured under iron deficiency |
| Iron low |  |
| Low B12 |  |
| Low ferritin |  |
| Low hgb |  |
| Low hemoglobin |  |
| Low iron |  |
| Vitamin B12 deficiency | captured under b12 deficiency |
| Vitamin b12 injection | captured under b12 injection |
| Vit B12 injection | captured under b12 injection |
| Vitamin B12 replacement therapy |  |
| Vit b12 shot | captured under b12 shot |

**Arthritis**

| **Free Text Term** | **Notes** |
| --- | --- |
| Arthritis |  |
| Arthralgia |  |
| Arthritis chronic | captured under arthritis |
| Arthritis gout | captured under arthritis |
| Arthritis psoriatic | captured under arthritis |
| Arthritis rheumatoid | captured under arthritis |
| arthritis septic | captured under arthritis |
| arthritis inflammatory | captured under arthritis |
| Arthropathy |  |
| Arthritic |  |
| Gout |  |
| Gouty | captured under gout |
| Gouty arthropathy | captured under arthropathy and gout |
| Hip replacement |  |
| Joint pain |  |
| Knee replacement |  |
| Osteoarthritis | captured under arthritis |
| Pain in joint |  |
| Polyarthralgia | captured under arthralgia |
| Polyarthritis | captured under arthritis |
| Pseudogout | captured under gout |
| Rheumatoid arthritis | captured under arthritis |
| Spondylopathy |  |
| *note: acronym OA not included as it is too broad and captures words like "throat" | |

**Atrial Fibrillation**

| **Free Text Term** | **Notes** |
| --- | --- |
| A fib |  |
| Abnormal ECG |  |
| Abnormal electrocardiogram |  |
| AFib |  |
| Arrythmia |  |
| Atrial fib |  |
| Atrial Fibrillation | captured under atrial fib |
| Atrial flutter | captured under flutter |
| Cardiac dysrhythmia |  |
| ECG abnormal |  |
| EKG abnormal |  |
| Flutter | exclusion: ventricular |
| Heart flutter | captured under flutter |
| Heart fluttering | captured under flutter |
| Heart rhythm irregular |  |
| Irregular heartbeat |  |
| PAF |  |
| Paroxysmal atrial fibrillation |  |

**Cerebrovascular Disease**

| **Free Text Term** | **Notes** |
| --- | --- |
| Brain aneurysm |  |
| Brain ischemia |  |
| Cerebellar atrophy |  |
| Cerebellar CVA |  |
| Cerebral aneurysm |  |
| Cerebral artery occlusion |  |
| Cerebral CVA |  |
| Cerebral degeneration |  |
| Cerebral infarction |  |
| Cerebral thrombosis |  |
| Cerebrovascular accident |  |
| Cerebrovascular disease |  |
| CVA |  |
| Intracerebral haemorrhage |  |
| Intracerebral hemorrhage |  |
| Intracranial aneurysm |  |
| Intracranial bleed |  |
| Lacunar |  |
| Mini stroke | captured under stroke |
| Post stroke | captured under stroke |
| Post CVA | captured under CVA |
| Post TIA |  |
| Stroke |  |
| Stroke clinic | captured under stroke |
| Stroke recovery | captured under stroke |
| Subarachnoid hemorrhage |  |
| Subarachnoid haemorrhage |  |
| Subdural hematoma |  |
| Subdural hemorrhage |  |
| Subdural haemorrhage |  |
| Transient ischemic attack |  |
| *Note: acronym TIA not included as it is too broad and captures words like "essential" | |
| *Note: acronym CVD not included as the terms often refer to screening for cardiovascular disease as opposed to a diagnosis | |

**Chronic Kidney Disease**

| **Free Text Term** | **Notes** |
| --- | --- |
| Chronic kidney disease |  |
| CKD |  |
| CRF |  |
| Chronic renal failure |  |
| End stage renal disease | captured under renal disease |
| Microalbuminuria |  |
| Nephrology |  |
| Nephropathy |  |
| Nephrotic syndrome |  |
| Proteinuria |  |
| Renal disease |  |
| Renal disease chronic | captured under renal disease |
| Renal disease end stage | captured under renal disease |
| Renal failure |  |
| Renal insufficiency |  |
| Uremia |  |

**Diabetes**

| **Free Text Term** | **Notes** |
| --- | --- |
| BG management |  |
| Blood sugar elevated |  |
| DM | exclusions: adm, abdm, cdm, sdm |
| CDM GV | captured under DM |
| Diabetic |  |
| Diabetic foot | captured under diabetic |
| Diabetic foot ulcer | captured under diabetic |
| Diabetic ketoacidosis | captured under diabetic |
| Diabetic neuropathy | captured under diabetic |
| diabetic ulcer | captured under diabetic |
| Diabetes |  |
| Diabetes Insipidus | captured under diabetes |
| Diabetes mellitus | captured under diabetes |
| DM2 | captured under DM |
| DMGV | captured under DM |
| DM Group Visit | captured under DM |
| DM Review | captured under DM |
| hyperglycemia |  |
| hypoglycemia |  |
| IFG |  |
| Impaired fasting glucose |  |
| impaired glucose tolerance |  |
| Insulin |  |
| Low blood sugar |  |
| NIDDM | captured under DM |
| IDDM | captured under DM |
| T2 Diabetes | captured under diabetes |

**Dizziness**

| **Free Text Term** | **Notes** |
| --- | --- |
| Dizziness |  |
| Dizzyness | captured under dizzy |
| Dizzy |  |
| Dizziness chronic | captured under dizziness |
| Dizziness intermittent | captured under dizziness |
| Dizzy spell | captured under dizzy |
| Epidemic vertigo | captured under vertigo |
| Peripheral vertigo | captured under vertigo |
| Vertigo |  |

**Dyspnea**

| **Free Text Term** | **Notes** |
| --- | --- |
| Breathing difficulty | captured under new term "breathing difficult" |
| Breathing difficulties | captured under new term "breathing difficult" |
| Breathing issue |  |
| Breathing problem |  |
| Dyspnea on exertion | captured under dyspnea |
| Dyspnea |  |
| Dyspnoea |  |
| Dyspneic |  |
| Difficult breathing |  |
| Difficulty breathing |  |
| SOB |  |
| Short of breath |  |
| Shortness of breath |  |
| shortness of breath on exertion | captured under shortness of breath |
| SOBOE | captured under SOB |
| Trouble breathing |  |

**Falls**

| **Free Text Term** | **Notes** |
| --- | --- |
| Fall | Exclusion: "Fallopian" |
| Falling | captured under fall |
| Falls | captured under fall |
| Fall injury | captured under fall |
| Fall injuries | captured under fall |
| Fall multiple | captured under fall |
| Fall related injury | captured under fall |
| Fall risk | captured under fall |
| Fell |  |
| Fell down | captured under fell |
| Frequent falling | captured under fall |
| Frequent falls | captured under fall |
| History of fall | captured under fall |
| Hx fall | captured under fall |
| Orthostatic fall | captured under fall |
| Recent fall | captured under fall |
| Recurrent falls | captured under fall |
| Risk of falling | captured under fall |
| Risk of fall | captured under fall |
| Unwitnessed fall | captured under fall |
| Witnessed fall | captured under fall |
| *Note: exclusion criteria for "falls asleep" applied | |

**Foot Problems**

| **Free Text Term** | **Notes** |
| --- | --- |
| Bunion |  |
| Callous on foot |  |
| Corns |  |
| Flat feet |  |
| Flat foot |  |
| Feet concern |  |
| Foot abnormality |  |
| Foot callous |  |
| Foot care |  |
| Foot concern |  |
| Foot exam |  |
| Foot gangrene |  |
| Foot infection |  |
| Foot issue |  |
| Foot lesion |  |
| Foot pain |  |
| Foot problem |  |
| Foot wound |  |
| Heel pain |  |
| Ingrown toenail |  |
| Infected toe |  |
| Orthotics |  |
| Orthotics form | captured under orthotics |
| Plantar fasciitis |  |
| Plantar wart |  |
| Podiatry |  |
| Podiatry referral | captured under podiatry |
| Podiatrist |  |
| Toe callous |  |
| Toe infection |  |
| Toenail infection |  |
| Toenail fungus |  |
| Toenail care |  |
| Wound on foot |  |

**Fragility Fracture**

| **Free Text Term** | **Notes** |
| --- | --- |
| Broken | Exclusion: broken teeth/tooth |
| Cast |  |
| Fracture |  |
| Fractured | captured under fracture |
| Stress fracture | captured under fracture |
| # | Exclusion: terms related to vaccine, injection, tpi, pain, and vax # |

**Hearing Impairment**

| **Free Text Term** | **Notes** |
| --- | --- |
| Audiometry |  |
| Can’t hear |  |
| Cochlear implant |  |
| Deaf |  |
| Deafness | captured under deaf |
| Decreased hearing |  |
| Hard of hearing |  |
| Hearing aid |  |
| Hearing decreased |  |
| Hearing deficit |  |
| Hearing impaired |  |
| Hearing impairment |  |
| Hearing issue |  |
| Hearing loss |  |
| Hearing problem |  |
| Presbyscusis |  |
| Reduced hearing |  |
| Trouble hearing |  |

**Heart Failure**

| **Free Text Term** | **Notes** |
| --- | --- |
| CHF |  |
| Congestive heart failure | captured under heart failure |
| Heart failure |  |

**Heart Valve Disease**

| **Free Text Term** | **Notes** |
| --- | --- |
| Aortic regurgitation |  |
| Aortic stenosis |  |
| Mitral valve prolapse |  |
| Mitral regurgitation |  |
| Mitral valve disorder | captured under valve disorder |
| Prosthetic aortic valve |  |
| Prosthetic mitral valve |  |
| Stenosis of aortic valve |  |
| Tricuspid regurgitation |  |
| Valvular heart disease |  |
| Valve disease |  |
| Valve disorder |  |
| Valve stenosis |  |

**Housebound**

| **Free Text Term** | **Notes** |
| --- | --- |
| Bed bound |  |
| Bedridden |  |
| Bed ridden |  |
| Home and community care |  |
| Home and community care referral | captured under home and community care |
| Home care |  |
| Home care issue | captured under home care |
| Home care need | captured under home care |
| Home care referral | captured under home care |
| Home health |  |
| Home support |  |
| Home visit | Exclusion: care home visit, nursing home visit |
| House call |  |
| House visit |  |

**Hypertension**

| **Free Text Term** | **Notes** |
| --- | --- |
| Blood pressure med |  |
| Blood pressure medication | captured under blood pressure med |
| BP rise |  |
| Elevated BP |  |
| Elevated blood pressure |  |
| HTN | Exclusion: "ghtn' |
| HTN Group | captured under HTN |
| HTN GV | captured under HTN |
| Hypertension |  |

**Hypotension/Syncope**

| **Free Text Term** | **Notes** |
| --- | --- |
| Blood pressure low |  |
| BP low |  |
| Cardiac syncope | captured under syncope |
| Faint |  |
| Fainting | captured under fainting |
| hypotension | Captured under new term "hypotensi" |
| hypotension chronic | Captured under new term "hypotensi" |
| hypotension orthostatic | Captured under new term "hypotensi" |
| hypotension persistent | Captured under new term "hypotensi" |
| hypotensive | Captured under new term "hypotensi" |
| Hypotensive episode | Captured under new term "hypotensi" |
| Lightheaded |  |
| Lightheadedness | captured under lightheaded |
| Low bp |  |
| Orthopnea |  |
| Orthosis |  |
| Orthostasis | captured under new term "orthosta" |
| Orthostatic | captured under new term "orthosta" |
| Orthostatic hypotension | captured under orthostatic |
| Passing out |  |
| Postural hypotension | captured under hypotension |
| Postural lightheadedness | captured under lightheaded |
| Presyncope | captured under syncope |
| Pseudo-syncope | captured under syncope |
| Syncopal episode |  |
| Syncope |  |
| Syncope micturition | captured under syncope |
| Syncope recurrent | captured under syncope |
| Syncope syndrome | captured under syncope |
| Vasovagal episode |  |

**Ischemic Heart Disease**

| **Free Text Term** | **Notes** |
| --- | --- |
| Angina |  |
| ASHD |  |
| Atherosclerotic heart disease | captured under heart disease |
| Atypical chest pain | captured under chest pain |
| Cardiovascular disease |  |
| Chest discomfort |  |
| Chest heaviness |  |
| Chest pain |  |
| Chest pressure |  |
| Chest tight |  |
| Coronary artery disease |  |
| Coronary atherosclerosis |  |
| CVD |  |
| Heart disease |  |
| Heart attack |  |
| Ischemic heart disease | captured under heart disease |
| Ischaemic heart disease | captured under heart disease |
| IHD |  |
| Mini heart attack | captured under heart attack |
| Myocardial Infarction |  |
| NSTEMI | captured under STEMI |
| STEMI | Exclsusions: systemic, Stemitil |
| Post MI |  |
| Recent MI |  |
| Tight chest |  |

**Memory and/or Cognitive Problems**

| **Free Text Term** | **Notes** |
| --- | --- |
| Alzheimer |  |
| Alzheimer’s Disease | captured under Alzheimer |
| Cognition | Exclsusion: normal cognition |
| Cognitive concern |  |
| Cognitive deficit |  |
| Cognitive decline |  |
| Cognitive impairment |  |
| Cognitive issue |  |
| Cognitive loss |  |
| Concern about memory | captured under memory |
| Confusion |  |
| Dementia |  |
| Early memory loss | captured under memory |
| Memory |  |
| Memory change | captured under memory |
| Memory concern | captured under memory |
| Memory decline | captured under memory |
| Memory impairment | captured under memory |
| Memory issue | captured under memory |
| Memory lapse | captured under memory |
| Memory loss | captured under memory |
| Memory loss issue | captured under memory |
| Memory problem | captured under memory |
| Mild cognitive impairment | captured under cognitive impairment |
| Neurocognitive impairment | captured under cognitive impairment |

**Mobility and/or Transfer Problems**

| **Free Text Term** | **Notes** |
| --- | --- |
| Abnormality of gait | captured under gait |
| Ataxia |  |
| Balance difficulty |  |
| Balance issue |  |
| Balance problem |  |
| Decreased mobility |  |
| Difficulty moving |  |
| Dysmobility |  |
| Gait |  |
| Gait abnormal | captured under gait |
| Gait change | captured under gait |
| Gait unstable | captured under gait |
| Gait unsteady | captured under gait |
| Handidart |  |
| Handydart |  |
| Hemiplegia |  |
| Immobility |  |
| Impaired mobility |  |
| Mobility concern |  |
| Mobility issue |  |
| Mobility problem |  |
| Paralysis |  |
| Paralysis partial | captured under paralysis |
| Paraplegia |  |
| Poor mobility |  |
| Poor balance |  |
| Problem with balance |  |
| Quadriplegia |  |
| Scooter |  |
| Unsteady |  |
| Unsteadiness |  |
| Unsteady gait | captured under gait |
| Walker |  |
| Wheelchair |  |

**Osteoporosis**

| **Free Text Term** | **Notes** |
| --- | --- |
| Low bone density |  |
| Low bone mass |  |
| Osteopenia |  |
| Osteoporosis | Also added term with 0 (zero) for spelling |
| Osteomalacia |  |
| *Note: acronym OP not included as it is too broad and captures several other words | |

**Parkinsonism and Tremor**

| **Free Text Term** | **Notes** |
| --- | --- |
| Benign essential tremor | captured under tremor |
| Extrapyramidal |  |
| Hand tremor | captured under tremor |
| Idiopathic tremor | captured under tremor |
| Idiopathic tremour | captured under tremor |
| Parkinson |  |
| Parkinsonism | captured under Parkinson |
| Tremor |  |
| Tremour |  |
| Tremulousness |  |

**Peptic Ulcer**

| **Free Text Term** | **Notes** |
| --- | --- |
| Blood in stool |  |
| Bloody stool |  |
| Colon ulcer |  |
| Duodenal ulcer |  |
| Esophageal ulcer |  |
| Gastric ulcer |  |
| Gastrointestinal bleed |  |
| Gastrointestinal haemorrhage |  |
| Gastrointestinal hemorrhage |  |
| GI bleed |  |
| H pylori | captured under new term "h%pylori" |
| H. Pylori | captured under new term "h%pylori" |
| Helicobacter pylori |  |
| Occult blood |  |
| Peptic ulcer |  |
| peptic ulcer disease | captured under peptic ulcer |
| PUD |  |
| Stool heme positive |  |

**Peripheral Vascular Disease**

| **Free Text Term** | **Notes** |
| --- | --- |
| Arteriosclerosis |  |
| Arterial disease |  |
| Arterial insufficiency |  |
| Atherosclerosis | except coronary atherosclerosis |
| Blood clot |  |
| Deep vein thrombosis |  |
| DVT |  |
| Embolism | excluding cerebral embolism |
| Lower extremity arterial insufficiency | captured under arterial insufficiency |
| Lower extremity thrombophlebitis |  |
| Lower extremity venous insufficiency |  |
| Lower extremity venous stasis | captured under venous stasis |
| Lower leg venous stasis | captured under venous stasis |
| Peripheral vascular disease |  |
| PVD |  |
| Thromboembolic disease |  |
| Thrombosis | excluding cerebral thrombosis |
| vascular deficiency |  |
| Vascular disorder |  |
| Vascular insufficiency |  |
| Venous stasis |  |
| Venous thrombosis | captured under thrombosis |

**Polypharmacy**

n/a

**Requirement for Care**

| **Free Text Term** | **Notes** |
| --- | --- |
| ADLs |  |
| Banking |  |
| Errands |  |
| Grocery | captured under new term "grocer" |
| Groceries | captured under new term "grocer" |
| IADLs | captured under ADLs |
| Lifeline |  |
| No help |  |
| Needs help |  |
| Palliative care |  |

**Respiratory Disease**

| **Free Text Term** | **Notes** |
| --- | --- |
| Asthma |  |
| Bronchiectasis |  |
| Bronchitis |  |
| Chronic airway obstruction |  |
| Chronic cough |  |
| Chronic lung disease | captured under lung disease |
| Chronic respiratory condition |  |
| Consistent cough |  |
| COPD |  |
| Cough chronic |  |
| CPAP |  |
| Emphysema |  |
| Home O2 |  |
| Home oxygen |  |
| Inhaler |  |
| Interstitial lung disease | captured under lung disease |
| Low oxygen |  |
| Lung disease |  |
| Nebulizer |  |
| 02 saturation low |  |
| oxygen saturation low |  |
| Ongoing cough |  |
| Persistent cough |  |
| Pulmonary edema |  |
| Pulmonary embolism |  |
| Pulmonary heart disease |  |
| Respiratory difficulty |  |
| Respiratory distress |  |
| Spirometry |  |
| *Note: acronym PE not included as it is too broad and captures several other words | |

**Skin Ulcer**

| **Free Text Term** | **Notes** |
| --- | --- |
| Bedsore |  |
| Chronic skin ulcer | captured under skin ulcer |
| Chronic ulcer |  |
| Decubitus ulcer |  |
| Open wound |  |
| Poor wound healing |  |
| Pressure sore |  |
| Pressure ulcer |  |
| Pressure wound |  |
| Skin eruption |  |
| Skin ulcer |  |
| Ulcer decubitus |  |
| Wound care |  |
| Wound chronic |  |

**Sleep Disturbance**

| **Free Text Term** | **Notes** |
| --- | --- |
| Can’t sleep |  |
| Disturbed sleep |  |
| Insomnia |  |
| Lack of sleep |  |
| Night terror |  |
| Night time wandering |  |
| Not sleeping |  |
| Obstructive sleep apnea | captured under sleep apnea |
| Persistent insomnia | captured under insomnia |
| Poor sleep |  |
| Sleep aid |  |
| Sleep apnea |  |
| Sleep clinic |  |
| Sleep deprivation |  |
| Sleep diary |  |
| Sleep difficulty |  |
| Sleep disorder |  |
| Sleep disruption |  |
| Sleep disturbance |  |
| Sleep hygiene |  |
| Sleep issue |  |
| Sleep medication |  |
| Sleep problem |  |
| Sleep walking |  |
| Sleeping disorder |  |
| Sleeping issue |  |
| Sleeping medication |  |
| Sleeping pill |  |
| Sleepless |  |
| Sleeplessness | captured under sleepless |
| Trouble sleeping |  |
| Unable to sleep |  |
| *Note: acronym OSA not included as it is too broad and captures several other words | |

**Social Vulnerability**

| **Free Text Term** | **Notes** |
| --- | --- |
| Advocate for housing | captured under housing |
| Bereavement |  |
| Bereavement counselling | captured under bereavement |
| Care for spouse |  |
| Caregiver burnout |  |
| Caregiver burden |  |
| Caregiver stress |  |
| Death in family |  |
| Death of husband |  |
| Death of spouse |  |
| Death of wife |  |
| Economic problem |  |
| Elder abuse |  |
| Elderly neglect |  |
| Emotional support |  |
| Emotional issue |  |
| Family concern |  |
| Family conflict |  |
| Family crisis |  |
| Family death |  |
| Family distress |  |
| Family issue |  |
| Family problem |  |
| Family stressor |  |
| Family situation stressful |  |
| Financial problem |  |
| Grief |  |
| Grief reaction | captured under grief |
| homeless |  |
| housing |  |
| Housing note | captured under housing |
| Housing issue | captured under housing |
| Housing inquiries | captured under housing |
| Husband’s death |  |
| Intimate partner violence |  |
| Life stressor |  |
| Loneliness |  |
| Low mood |  |
| Marital conflict |  |
| Marital separation |  |
| Marital problem |  |
| No MSP |  |
| Poverty |  |
| Psychosocial problem |  |
| Psychological stress |  |
| Psychosocial Stressor |  |
| Psychosocial trauma |  |
| Psychological trauma |  |
| Relationship problem |  |
| Safety at home |  |
| Self neglect |  |
| Situational crisis |  |
| Social assistance |  |
| Social circumstance |  |
| Social isolation |  |
| Social issue |  |
| Social prescribing |  |
| Social problem |  |
| Social situation |  |
| Social work |  |
| Supplemental housing form | captured under housing |
| SW support |  |
| Transient housing | captured under housing |
| Unable to cope |  |
| Unstable housing | captured under housing |
| Wife’s death |  |
| *Note: acronym SW not included as it is too broad and captures several other words | |

**Thyroid Disorder**

| **Free Text Term** | **Notes** |
| --- | --- |
| Goiter |  |
| Hyperthyroid |  |
| Hyperthyroidism | captured under hyperthyroid |
| Hyperactive thyroid |  |
| Hypothyroid |  |
| Hypothyroidism | captured under hypothyroid |
| Low thyroid |  |
| Thyroid deficiency |  |
| Thyroid disorder |  |
| Thyroid function test abnormal |  |
| Thyroid goiter | captured under goiter |
| Thyroid issue |  |
| Thyroid nodule |  |
| Thyroiditis |  |

**Urinary Incontinence**

| **Free Text Term** | **Notes** |
| --- | --- |
| Atony of bladder |  |
| Bladder atony |  |
| Bladder control |  |
| Incontinence | Exclsusions: faecal, fecal, feces, faeces |
| Incontinence of urine | captured under incontinence |
| Mixed incontinence | captured under incontinence |
| Stress incontinence | captured under incontinence |
| Urge incontinence | captured under incontinence |
| Urinary incontinence | captured under incontinence |
| Urinary incontinence chronic | captured under incontinence |
| Urinary incontinence stress | captured under incontinence |
| Urinary incontinence overflow | captured under incontinence |
| Urinary incontinence urge | captured under incontinence |
| Urine incontinence | captured under incontinence |

**Urinary System Disease**

| **Free Text Term** | **Notes** |
| --- | --- |
| Benign prostate hyperplasia | captured under new term "%benign prostat% hyper%" |
| Benign prostatic hyperplasia | captured under new term "%benign prostat% hyper%" |
| Benign prostatic hypertrophy | captured under new term "%benign prostat% hyper%" |
| Bladder disorder |  |
| Bladder hypersensitivity |  |
| Bladder infection |  |
| Bladder issue |  |
| Bladder spasm |  |
| Blood in urine |  |
| BPH |  |
| Burning on voiding |  |
| Chronic UTI | captured under UTI |
| Cystitis | Exclsusions: terms related to cholecystitis |
| Cystitis recurrent | captured under cystitis |
| Difficulty urinating |  |
| Dysuria |  |
| Enlarged prostate |  |
| Elevated PSA |  |
| Elevated prostate specific antigen |  |
| Frequent urination |  |
| Haematuria |  |
| Hematuria |  |
| Lower urinary tract symptom |  |
| LUTS |  |
| Nocturia |  |
| Overactive bladder |  |
| Polyuria |  |
| Prostate abnormal |  |
| Prostate hyperplasia benign |  |
| Prostatism |  |
| Prostatitis |  |
| PSA elevated | captured under new term "PSA elevat" |
| Retention of urine |  |
| Recurrent UTI | captured under UTI |
| Urinalysis abnormal |  |
| Urine culture positive |  |
| Urine dipstick abnormal |  |
| Urinary problem |  |
| Urinary difficulty |  |
| Urinary frequency |  |
| Urinary hesitancy | captured under new term "urinary hesitanc" |
| Urinary issue |  |
| Urinary retention |  |
| Urinary symptoms |  |
| Urinary tract issue |  |
| Urinary tract infection |  |
| Urinary tract infection recurrent | captured under urinary tract infection |
| Urinary urgency |  |
| Urine retention |  |
| Urinary retention |  |
| UTI | Exclsusions: utic, utid, utin, utio, utis |
| UTI symptom | captured under UTI |

**Visual Impairment**

| **Free Text Term** | **Notes** |
| --- | --- |
| Amaurosis fugax |  |
| Blindness |  |
| Blurred vision |  |
| Blurry eyes |  |
| Blurry vision |  |
| Cataract |  |
| Deteriorating vision |  |
| Double vision |  |
| Early cataract | captured under cataract |
| Eye blurred | captured under new term "eye blurr" |
| Eye blurry | captured under new term "eye blurr" |
| Eye vision change | captured under vision change |
| Eyesight blurry |  |
| Hemianopsia |  |
| hyperopia |  |
| Low vision |  |
| Macular degeneration |  |
| Poor eyesight |  |
| Retinopathy |  |
| Sudden vision loss | captured under vision loss |
| Trouble with vision |  |
| Vision blurred | captured under new term "vision blurr" |
| Vision change |  |
| Vision deteriorating |  |
| Vision difficulties |  |
| Vision issues |  |
| Vision loss |  |
| Vision problem |  |
| Visual abnormalities |  |
| Visual acuity decreased |  |
| Visual disturbance |  |
| Visual field defect |  |
| Visual impairment |  |

**Weight Loss and/or Anorexia**

| **Free Text Term** | **Notes** |
| --- | --- |
| Acute weight loss | captured under weight loss |
| Anorexia |  |
| Anorexia nervosa | captured under anorexia |
| Appetite decreased |  |
| Lost weight |  |
| Malnourished |  |
| Malnutrition |  |
| Malnutrition chronic | captured under malnutrition |
| No appetite |  |
| Poor appetite |  |
| Poor nutrition |  |
| Underweight |  |
| Unexplained weight loss | captured under weight loss |
| Unwanted weight loss | captured under weight loss |
| Weight loss |  |
| Weight loss abnormal | captured under weight loss |
| Wt loss |  |
