# Supplementary material for: Development and testing of an electronic frailty index using Canadian electronic medical record data in primary care
Source: BMC Prim Care. 2025 Nov 12;26:359. doi: 10.1186/s12875-025-03075-7 (PMC12613384; doi:10.1186/s12875-025-03075-7)
Supplement: Supplementary file 3 — Supplementary Material 3. [file 12875_2025_3075_MOESM3_ESM.docx]

**Concurrent Polypharmacy**

This document provides a description of how we identified which patients met the criteria for concurrent polypharmacy.

Because we did not have a specific query that can identify which patients meet the concurrent polypharmacy definition in a large dataset, additional steps were required using Python Software and data available from BC-CPCSSN.

1. We found all patients with 5 or more medications prescriptions documented in the past 12 months (2021-07-01 to 2022-06-30).
2. We searched for the patients in the list above with a duration defined (either a valid stop date or a valid duration count) and found patients with 5+ medications with a defined duration.
3. We calculated the number of days since 2021-07-01 that the prescription started and ended.
4. For each patient and each medication, we populated a matrix of length 365, inserting a 1 for those days that covered the duration of a prescription (0 otherwise) with rows corresponding to each medication.
5. We calculated the sum of each column in the matrix.
6. We determined which patients had at least one day with a value of 5 or more in their vector.

We removed medications with no associated duration recorded. Duplicate prescriptions were irrelevant as they would overlap. Prescriptions that did not have an ATC code were included as separate prescriptions under the assumption that it is unlikely to have two prescriptions for the same medication over the same time period. Finally, the 12 month cut-off for the start date of the prescriptions that started before 2021-07-01 but extended into the 12-month period of interest, could lead to an underestimate of polypharmacy.
